# Rapid sol–gel synthesis of honeycomb-layered Na_3_Ni_2_BiO_6_ and orthorhombic Na_3_Ca_2_BiO_6_

**DOI:** 10.1039/d2dt04052b

**Published:** 2023-02-13

**Authors:** Emily J. Luke, Jason Potticary, Sven Friedemann, Simon R. Hall

**Affiliations:** a School of Chemistry, University of Bristol Cantock's Close Bristol BS8 1TS UK simon.hall@bristol.ac.uk; b School of Physics, HH Wills Physics Laboratory Tyndall Avenue Bristol BS8 1TL UK

## Abstract

The A_3_M_2_M′O_6_ type materials Na_3_Ca_2_BiO_6_ and Na_3_Ni_2_BiO_6_ were successfully synthesised through two sol–gel techniques – a method based on a natural deep eutectic solvent, and a biopolymer-mediated synthesis. The materials were analysed using Scanning Electron Microscopy to determine if there was a difference in final morphology between the two methods, and it was found that the natural deep eutectic solvent method resulted in a more porous morphology. For both materials, the optimum dwell temperature was found to be 800 °C, which in the case of Na_3_Ca_2_BiO_6_ was a much less energy-intensive synthesis process than its seminal solid-state synthesis. Magnetic susceptibility measurements were undertaken on both materials. It was found that Na_3_Ca_2_BiO_6_ exhibits only weak, temperature independent paramagnetism. Na_3_Ni_2_BiO_6_ was found to be antiferromagnetic, with a Néel temperature of 12 K, in line with previously reported results.

## Abstract 2

We present a new synthesis of a material that could have potential as a cathode material for sodium-ion batteries, Na_3_Ni_2_BiO_6_. We also apply this synthesis to a relatively unexplored material, Na_3_Ca_2_BiO_6_ and investigate its magnetic properties.

## Graphical abstract

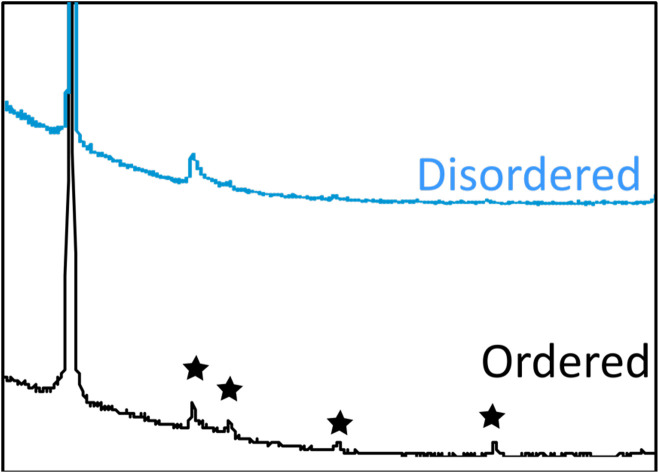

## Introduction

Crystalline materials based on α-NaFeO_2_, or more generally AMO_2_ where A is an alkali metal and M is a transition metal, are widely studied due to their potential applications as sodium-ion batteries (NIBs) and their interesting magnetic structure.^1–3^ With these materials, one-third of the M ions can be substituted for another element, forming a material with the formula A_3_M_2_M′O_6_, which commonly crystallises as one of two polymorphs depending on the relative sizes and charges of M and M′. When M and M′ are of similar ionic radius but of a significant difference in charge, a monoclinic polymorph forms, often referred to as a honeycomb-layered metal oxide. The other polymorph is a rock-salt type structure (space group – *Fddd*), with an orthorhombic unit cell, often referred to as the orthorhombic polymorph.^4^ It has been shown that these two polymorphs can be energetically similar enough that some materials are able to crystallise as either based on the synthetic method.^4^ Na_3_Ca_2_BiO_6_ (referred to hereafter as NCB) crystallises as the orthorhombic polymorph and is relatively unstudied. It was first synthesised in 2007 by Puzdrjakova *et al. via* a solid-state method.^5^ It was recently found that NCB formed instead of the superconductor Bi_2_Sr_2_CaCu_2_O_8_, when an attempt was made to synthesise the latter through a sol–gel method using sodium alginate as a chelating agent.^6^ In 2018, a machine learning algorithm identified NCB as a potential superconductor.^7^ As the magnetic and electronic properties of this material are hitherto unknown, the validity of this has yet to be verified.

Na_3_Ni_2_BiO_6_ (referred to hereafter as NNB) crystallises as a honeycomb layered metal oxide, where the spin-½ nickel ions form a hexagonal array around the bismuth ions interspersed between sodium layers. It has been well-explored due to its frustrated antiferromagnetism and potential as a NIB.^8–10^ NIBs provide tantalising potential alternatives to lithium-ion batteries (LIBs) due to the higher abundance of sodium in the Earth's crust, the higher relative safety of NIBs, and the similar chemistry of LIBs and NIBs enabling existing industrial and commercial infrastructure to be used.^11^ An isomorph of NNB, Na_3_Ni_2_SbO_6_, was shown to have excellent cycling stability and theoretical discharge capacity, indicating good potential as a NIB.^12,13^ Thus, NNB has also been explored for its potential as a cathode in NIBs and has shown promising results.^14^ One limitation of the A_3_M_2_M′O_6_ layered honeycomb materials, and by extension NNB, is the propensity to form stacking defects in the crystal structure. This leads to a decreased battery performance due to the disordered structure and manifests as so-called Warren peaks in the powder X-ray diffraction (PXRD) pattern.^15,16^ Warren peaks are characterised by a sharp drop of intensity at the low-angle range and a gradual decrease of intensity at higher angles.^17^ These peaks occur when a crystal lattice is not stacked correctly, for example with occasional layers of unit cells being out of alignment. This has implications for the intensity of the diffracted peak due to fewer lattice planes satisfying the Bragg condition and gives rise to the characteristic asymmetric shape.^18^ NNB was also predicted to be superconductive by the same machine learning algorithm that identified NCB as a potential superconductor. However, the magnetic behaviour of NNB has already been examined and appears not to be superconducting, but is instead antiferromagnetic.^7,19^ It was proposed in the machine learning study by Stanev *et al.*, that it is possible that doping the material could induce superconductivity, but this has also not been investigated experimentally.^7^

Both NCB and NNB are conventionally synthesised by a solid-state method, though a top-down synthesis of NNB *via* electrospinning has also seen some success.^20^ While the infrastructure behind solid-state syntheses is robust, the reaction times can be long, and there can be poor control of resultant morphology. These extended reaction times are due to slow mass transport in the solid state, necessitating long reaction times to ensure a phase pure product. In addition, further grinding and heating steps are also often utilised to ensure the reaction's completion. An alternative synthesis route is through a sol–gel method. In this case, the random mixing of the solution phase can overcome the limitations in mass transport.^21^ A potential synthetic route would be to use a biopolymer-mediated synthesis, where a biopolymer is added to an aqueous solution of the metal salts in stoichiometric amounts. Biopolymers behave as non-specific polychelating agents for metal cations, enabling the simultaneous chelation of a diverse range of cations in a single synthetic step.^22^ This method can result in good control of the nanostructure of the resulting metal oxide.^23^ These biopolymer-mediated reactions have been utilised previously to enable rapid syntheses of complex metal oxide materials and have also been shown to enable the control of the macro and nanostructure of the material.^24^

One downside of sol–gel techniques for synthesising bismuth-containing metal oxides is the poor solubility of bismuth nitrate in water. This is believed to be due to polycondensation reactions that occur between bismuth ions, resulting in the formation of bismuth subnitrate and other bismuth oxido clusters, which are insoluble.^25^ These solubility issues can be overcome in biopolymer-mediated approaches by adding chelating agents, such as ethylenediaminetetraacetic acid (EDTA).^26^ These chelating agents aid in the dissolution of the bismuth ions and prevent the formation of insoluble bismuth clusters. Following this addition, an aqueous solution of metal salts can be combined with a biopolymer for gelation. Another previously explored method for synthesising bismuth-containing metal oxides uses a natural deep eutectic solvent (NADES) containing betaine and d-(+)-glucose instead of water.^27^ Amine groups on the betaine molecules are able to chelate to the bismuth ions, such that in this synthesis the NADES can chelate to the constituent metal ions without the necessity for the addition of other chelating agents such as EDTA or biopolymers.

In this work, we present the synthesis of NCB and NNB *via* two different methods – a NADES-based synthesis and a biopolymer-mediated approach. We show that these methods result in high purity of product using Rietveld refinement, with much lower dwell times for both materials and lower synthesis temperatures in the case of NCB. By synthesising both NCB and NNB, we show that these two synthetic methods are compatible with both A_3_M_2_M′O_6_ polymorphs. Furthermore, for both materials, we perform SQUID magnetometry to examine their magnetic properties and Scanning Electron Microscopy (SEM) to investigate the nanostructure.

## Experimental

### Materials and methods

Nickel nitrate hexahydrate (98.5%), bismuth nitrate pentahydrate (98%), calcium nitrate tetrahydrate (≤99%), sodium nitrate (≤99.0%), ethylenediaminetetraacetic acid (≤98%), betaine (≤99.0%), d-(+)-glucose (≤99.5%), and dextran (*M*_r_ = 70 000) were purchased from Sigma Aldrich and used without any further purification.

#### NADES precursor solutions

Betaine (3.000 g) was added to d-(+)-glucose (1.845 g) and 6.336 mL of deionised water. The mixture was shaken until all powders had fully dissolved, yielding 10 mL of solution. The **Bi-NADES** precursor solution was made by adding Bi(NO_3_)_3_·5H_2_O (121.5 mg, 0.05 M) to 5 mL of the NADES solution and stirring until all solids had dissolved.

#### Aqueous precursor solutions

To generate the **NaCa-Aq** precursor solution, NaNO_3_ (131.5 mg, 0.15 M, 3% excess) and Ca(NO_3_)_2_·4H_2_O (232.6 mg, 0.10 M) were added to 10 mL of water, and the solution was stirred until all solids had dissolved. To generate the **NaNi-Aq** precursor solution, NaNO_3_ (131.5 mg, 0.15 M, 3% excess) and Ni(NO_3_)_2_·6H_2_O (290.1 mg, 0.10 M) were added to 10 mL water, and the solution stirred until all powders had dissolved. The **Bi-Aq** precursor solution was made by adding Bi(NO_3_)_3_·5H_2_O (242.5 mg, 0.05 M) to 10 mL of deionised water, with an additional 0.2 g ethylenediaminetetraacetic acid (EDTA) to aid its dissolution. This solution was stirred at 80 °C until all metal salts had dissolved. For the **NaNiBi-Aq** precursor solution, NaNO_3_ (131.5 mg, 0.15 M, 3%), Ni(NO_3_)_2_·6H_2_O (290.1 mg, 0.10 M), and Bi(NO_3_)_3_·5H_2_O (242.5 mg, 0.05 M) were added to 10 mL of water, along with 0.2 g EDTA. The solution was heated to 80 °C with stirring until all solids had dissolved.

### NADES synthesis method

For NCB, 1 mL Bi-NADES, 1 mL NaCa-Aq solution and 1.05 mL NADES were combined in a crucible before being heated to 80 °C for 2 hours to dehydrate the solution. This crucible was then calcined in a chamber furnace at 800 °C with a dwell time of 2 hours and a 5 °C min^−1^ ramp rate. For NNB, the same was carried out, substituting 1 mL of NaNi-Aq solution for the NaCa-Aq solution.

### Aqueous synthesis method

For NCB, 1 mL of the NaCa-Aq solution and 1 mL of the Bi-Aq was added to a crucible with 100 mg dextran and stirred mechanically until all lumps of material had dissolved. Then, the solution was allowed to dry overnight before being calcined in a chamber furnace at 800 °C with a dwell time of 2 hours and a 5 °C min^−1^ ramp rate. For NNB, 1 mL of the NaNiBi-Aq solution was added to a crucible with 100 mg dextran and stirred with a spatula until all lumps of material had dissolved. After drying overnight, this crucible was calcined with the same parameters as used for NCB.

### Characterisation

Powder X-ray diffraction (PXRD) was carried out on a Bruker D8 Advance with Cu-Kα, (*λ* = 1.5418 Å) source and a position-sensitive LynxEye Detector. Rietveld refinement was carried out with the open-source software Profex, which is a graphical interface for the refinement software, BGMN.^28^ Scanning electron microscopy was carried out on a JEOL IT300. Magnetic susceptibility measurements for NCB were carried out on a Quantum Design MPMS 3 magnetometer. Magnetic susceptibility measurements for NNB were carried out by the Henry Royce Institute at Sheffield, also on a Quantum Design MPMS 3. To calculate the volume susceptibility of the samples, the density of the material taken from the published crystal structure was used, combined with the mass of the sample to calculate the volume of material.

## Results and discussion

**Fig. 1:**
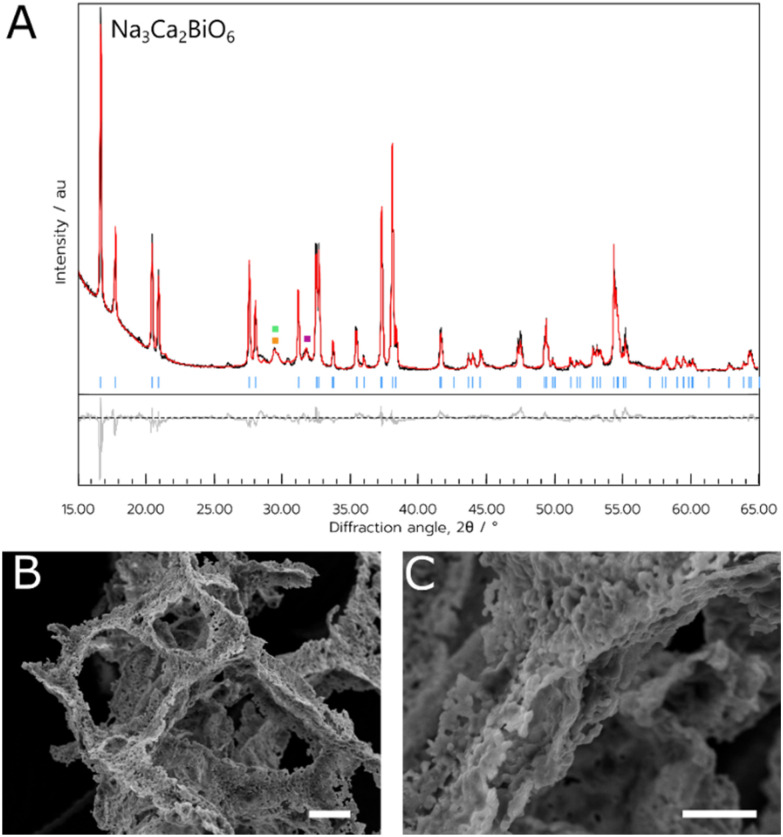
(A) PXRD data of the NCB NADES sample. Blue indices are attributed to the Bragg reflections of the target Na_3_Ca_2_BiO_6_. Impurity peaks belonging to Ca_4_Bi_6_O_13_, Bi_6_Ca_6_O_15_, and β-Bi_2_O_3_ are denoted by green, orange and purple squares respectively. (B) and (C) SEM images of the NCB NADES sample. Scale bars indicate a length of (B) 20 μm and (C) 10 μm.

For both materials, the NADES synthesis yielded the target phase. For NCB, the target phase was present when synthesised with a dwell temperature of 700, 800 or 900 °C. In this case, the optimal temperature appeared to be 800 °C (referred to hereafter as NCB-NADES), with a phase purity of 91% estimated by multi-phase Rietveld refinement (shown in Fig. 1). Some mixed Ca–Bi–O phases were also observed as impurity phases. These phases were likely kinetically favourable to form during the short synthesis. When calcined at 600 °C or 1000 °C, the target phase was not observed. This is interesting as the solid-state synthesis requires a dwell temperature of 1100 °C for 24 hours, followed by slow cooling to 800 °C and remaining there for 1 hour before cooling to room temperature.^5^ The NADES synthesis, on the other hand, only required a two-hour dwell time at elevated temperature to form the target phase. As such, it appears that by synthesising the material through this sol–gel method, the calcination times and temperature has been significantly lowered without compromising on the purity of the material. The polycrystalline orange powder appeared to exhibit a flaky, porous structure when examined with SEM. The pores could be due to the decomposition of the NADES into CO_2_ during the heating cycle, which forms bubbles within the remaining metal oxide.

**Fig. 2:**
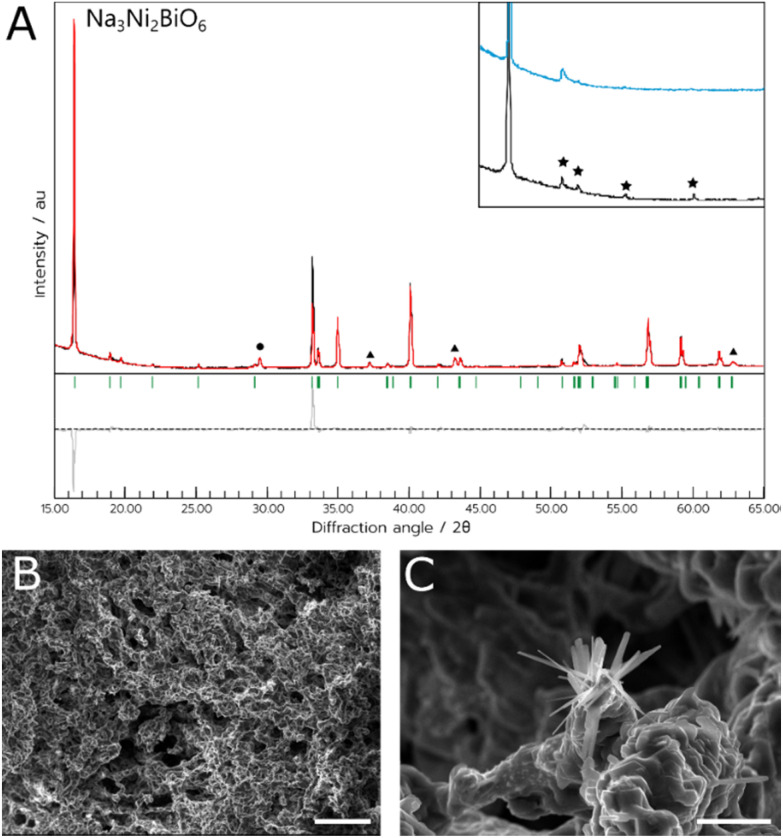
(A) PXRD data of the NNB-NADES sample. Green indices are attributed to the Bragg reflections of the target Na_3_Ni_2_BiO_6_ phase. Impurities of NiO and NaNO_3_ are indicated by the triangle and circle symbols, respectively. Inset shows a magnified section of the experimental data between 15° and 27° 2*θ* with stars denoting the peaks arising from the hexagonal superlattice. In blue is an example of an experiment that resulted in the Warren peak instead of peaks belonging to the hexagonal superlattice. (B) Scanning electron micrograph depicting the as-synthesised material. (C) Scanning electron micrograph depicting the as-synthesied material, focusing on the nanowire-like growths that occurred throughout. Scale bar in (B) indicates a length of 100 μm, (C) indicates a length of 10 μm.

In the case of NNB, the target phase also formed at temperatures of 800 or 900 °C, with an optimal temperature of 800 °C (referred to hereafter as NNB-NADES). As noted above, impurity phases of NiO and NaNO_2_ were also observed, This synthesis occasionally yielded a product with stacking defects in the crystal structure, evidenced by the lack of peaks attributed to the hexagonal superlattice of the material and the presence of a Warren peak instead, an example of this can be seen in the inset of Fig. 2A.^15^ The presence of this disordered polymorph of NNB is likely due to the variability of the atmosphere within the furnace, as NNB is a highly oxygenated material and the seminal synthesis required an atmosphere of flowing oxygen in an annealing step to yield the ordered polymorph of the target phase.^8^ While using an atmosphere of flowing oxygen would be incompatible with this synthesis due to the combustible precursors; further annealing steps could be utilised on this material to yield the ordered polymorph of the material. The ordered polymorph formed when the experiment was carried out in an open tube furnace, further indicating the necessity for atmospheric control for the synthesis. Additionally, the intensity of the (002) peak is relatively higher than would be expected compared to the intensity of the (001) peak, shown on the difference plot in Fig. 2A. We suggest that this could be due to differences in the occupancy of the Bi-ions when compared to the Ni-ions. It was observed in the initial study of NNB that there were issues calculating the occupancies of the Ni-ions and Bi-ions, which could be the source of the peak mismatch due to the difference in scattering factors of Bi and Ni.

The material's structure was predominantly porous, but there was also evidence of micro- and nanowires spread sporadically throughout the porous network, as shown in Fig. 4C. As with the NCB-NADES sample, the porous network appeared to be randomly orientated with an almost sponge-like morphology. The porous nature of this material could be beneficial for applications such as electrodes. It has been shown that a porous material for an electrode could be ideal, as the larger surface area could allow ample contact between the electrolyte and the electrode surface.^29^ Although the ordered polymorph did not always form, this could likely be rectified in the future by utilising additional annealing steps.

The other synthesis attempted was a biopolymer-mediated synthesis. This method utilised the biopolymer dextran as a chelating agent. As mentioned earlier, this is an aqueous synthetic method, an additional chelating agent of EDTA was required to assist the dissolution of the bismuth nitrate pentahydrate. For both materials, dwell temperatures from 600–1000 °C were used with 2-hour dwell times.

For the NCB, the bismuth nitrate was dissolved in a separate solution from the sodium nitrate and calcium nitrate. This was due to the propensity of the salts to precipitate out of solution when all dissolved together after several hours. However, when prepared separately, the metal salts remained dissolved for several days at least. Therefore, two aqueous solutions were made separately, one with the bismuth nitrate and the other with the calcium and sodium nitrates. These were then pipetted into the crucible and combined with the dextran before complete drying. The optimum temperature for synthesis of the target material was 800 °C (referred to hereafter as NCB-Aq), similar to the NADES method. Rietveld refinement performed on these samples suggested the product comprised of 79% of the target phase. A summary of the PXRD data for the NCB samples as determined by Rietveld refinement can be found in Table 1.

**Table 1:** The crystalline volume fraction data from the multi-phase Rietveld refinement for the two NCB samples discussed in this work and the entry code for the respective crystal structures in the Inorganic Crystal Structure Database

Phase	800 °C, NADES volume/%	800 °C, aqueous volume/%	ICSD number
Na_3_Ca_2_BiO_6_	91	79	240975
Bi_6_Ca_6_O_15_	3	3	73780
Ca_4_Bi_6_O_13_	3	3	68948
Bi_4_O_7_	3	—	51778
NaNO_3_	—	15	14185
*R*_wp_	7.8	9.2	—

**Fig. 3:**
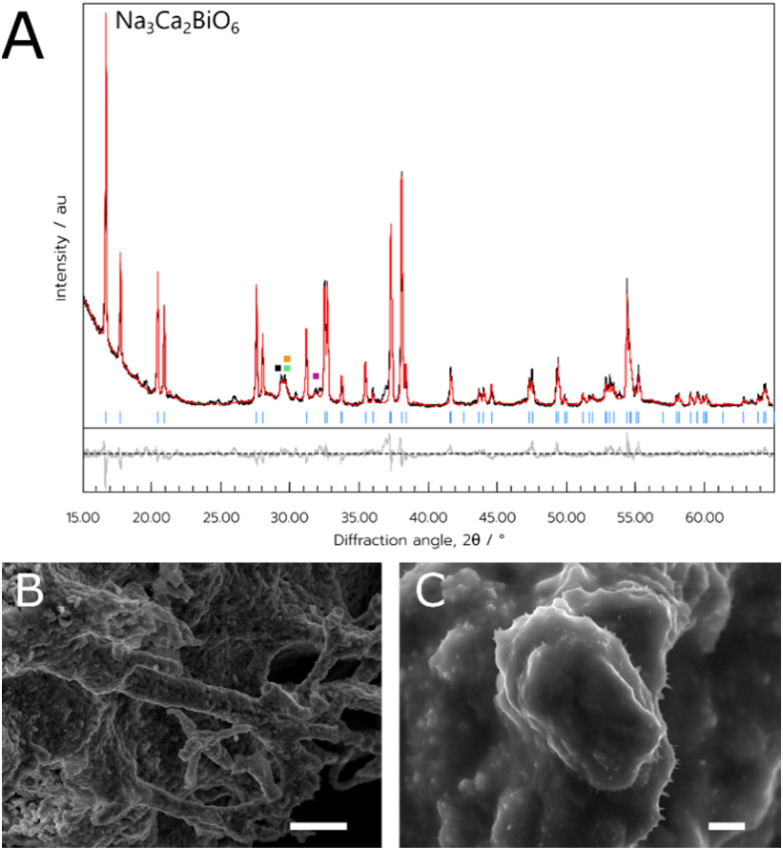
(A) PXRD data of the NCB sample synthesised through the aqueous method. Blue indices are attributed to the Bragg reflections of the target Na_3_Ca_2_BiO_6_. Impurity peaks belonging to Ca_4_Bi_6_O_13_, Bi_6_Ca_6_O_15_, and β-Bi_2_O_3_, NaNO_3_ are denoted by green, orange, purple and black squares respectively. (B) and (C) SEM images of the NCB-aqueous sample. Scale bars indicate a length of (B) 10 μm and (C) 2 μm.

Finally, the orange powder was examined with SEM (Fig. 3B and C), and the sample had a more amorphous consistency, with a lack of observable crystallites. Additionally, there were some very thin wire-like growths observed growing from the mass of material when examined at higher magnifications (Fig. 3C).

**Fig. 4:**
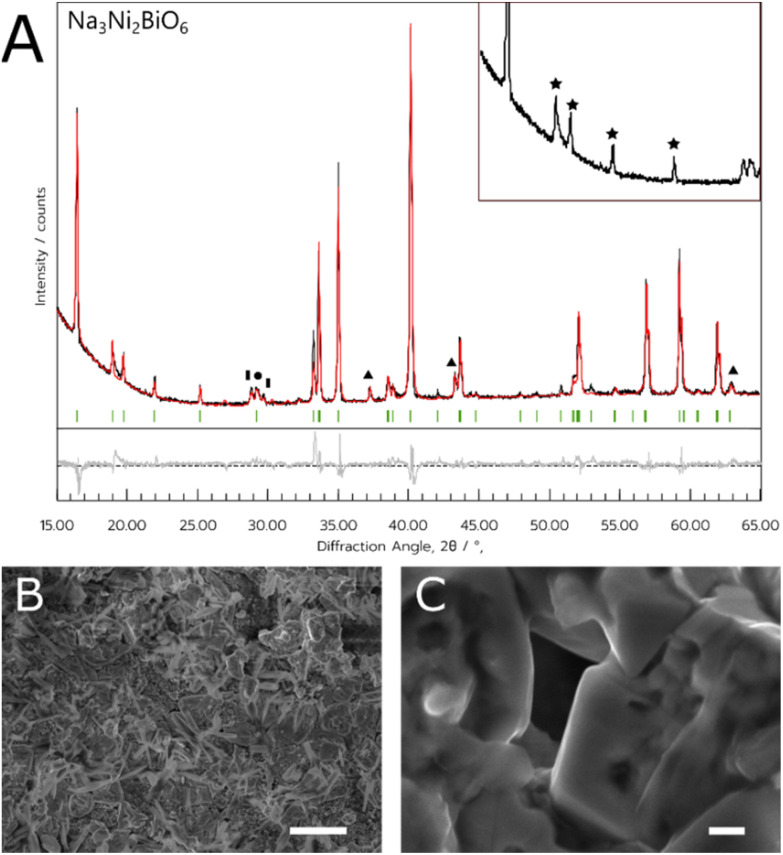
(A) PXRD data of the NNB sample synthesised using 100 mg dextran as the chelating agent. Green indices are attributed to the Bragg reflections of the target Na_3_Ni_2_BiO_6_ phase. Impurities of NiO and NaNO_3_ are indicated by the triangle and circle symbols, respectively. Inset shows a magnified section of the experimental data between 15° and 27° 2*θ* with stars denoting the peaks arising from the hexagonal superlattice. (B) and (C) SEM images of the NNB sample, depicting a clear difference in morphology of the sample. Scale bar in (B) indicates a length of 20 μm, (C) indicates a length of 1 μm.

This synthesis method with NNB appeared to be more successful than the equivalent method with NCB, as the metal salts were consistently able to dissolve. As with the NADES method, the optimum dwell temperature in this case was 800 °C (sample referred to hereafter as NNB-Aq). Additionally, the ordered NNB polymorph formed more consistently with this method, as seen by the higher intensity hexagonal superlattice peaks (Fig. 4a). The material was less porous than the NADES synthesised material, tending to form chunks of solid rather than a porous matrix. There was some evidence of the nanowire-like growths in this material, but the majority of the sample comprised blocky crystallites. A summary of the Rietveld refinement results for NNB can be found in Table 2.

**Table 2:** The crystalline volume fraction data from the multi-phase Rietveld refinement of the two NNB samples discussed in this work and the entry code for the respective crystal structures in the Inorganic Crystal Structure Database

Phase	800 °C, NADES volume/%	800 °C, aqueous volume/%	ICSD number
Na_3_Ni_2_BiO_6_	76	82	237391
NiO	7	6	24014
NaNO_3_	17	9	14185
Bi_2_O_3_	—	2	169686
*R*_wp_	9.8	5.6	

**Fig. 5:**
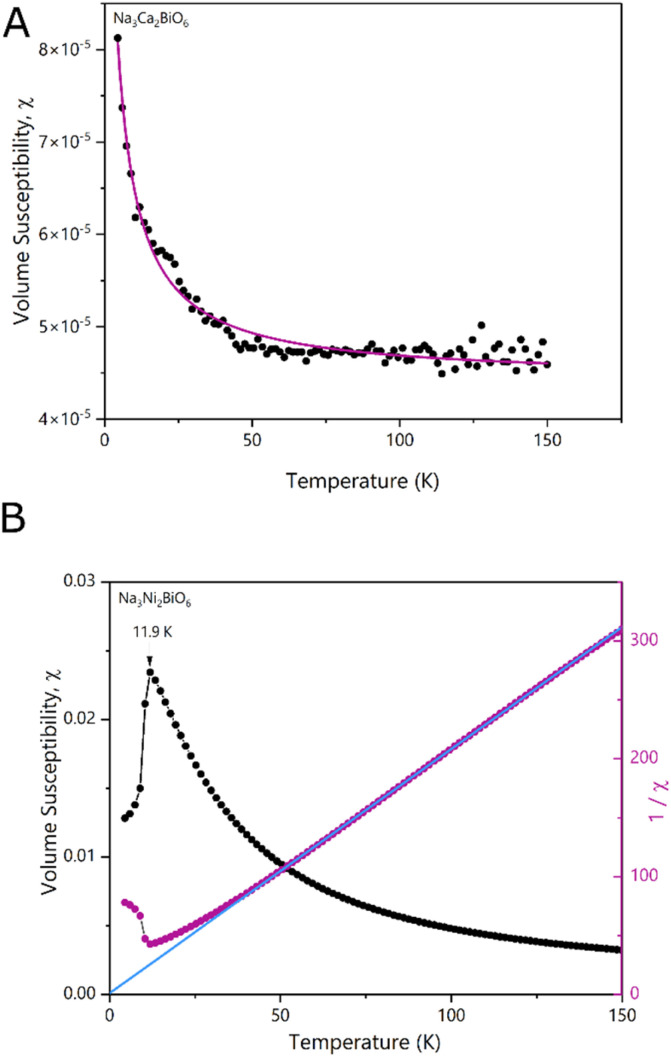
Zero-field cooled SQUID magnetometry data of the (A) NCB-NADES sample. The solid line is a fit to the Curie–Weiss law, 
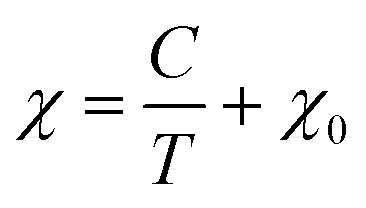
 (B) NNB-Aq sample. The black data points are associated with the left axis, while the purple data points are associated with the right axis. The solid blue line is a fit of the Curie–Weiss form 
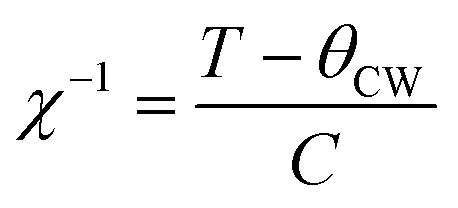
 to *χ*_0_. Both data are zero-field cooled plots, with an applied field of 100 Oe.

NCB-NADES was examined by SQUID magnetometry due to its higher purity than NCB-Aq. A characteristic upturn of magnetic susceptibility can be seen in the data (Fig. 5A), indicative of a paramagnetic material. However, the overall susceptibility is very small, indicating that the sample was only weakly paramagnetic. The temperature dependence *χ*(*T*) is well described by a Curie term in addition to a temperature independent susceptibility *χ*_0_ (solid line in Fig. 5A). The temperature independent term suggests weak van Vleck paramagnetism of Na_3_Ca_2_BiO_6_. The Curie term, however, is associated with contaminations. The Cure constant, 2.65 × 10^−4^ K^−1^ reflects a small concentration of ∼0.13% of magnetic ions such as iron, cobalt or nickel.

Due to the prevalence of the hexagonal superlattice peaks, SQUID magnetometry was carried out on NNB-Aq to affirm the magnetic properties. The sample exhibited a characteristic antiferromagnetic shape, with a Néel temperature of 11.9 K, which is in line with the literature reported value.^8^ Linear regression on *χ*^−1^(*T*) between 40–120 K shows that NNB exhibits Curie–Weiss behaviour. The Curie–Weiss temperature, *θ*_CW_, of the sample was found to be 0.79 K. A positive *θ*_CW_ combined with an antiferromagnetic transition are indicative of a frustrated system, though, in this case, the low magnitude of *θ*_CW_ when compared to that of the seminal paper indicates that the degree of frustration is low. Following the determination of the Curie constant, we were also able to calculate the effective moment, *μ*_eff_ of the Nickel ions within the crystal lattice to be 2.90*μ*_B_, which corresponds to a divalent state, as expected for NNB, and corresponds to the originally reported work.

## Conclusion

In this work, we have successfully synthesised NCB and NNB through two rapid methods; one utilising a NADES, and another *via* an aqueous sol–gel route utilising the biopolymer dextran as a chelating agent. Both methods drastically reduced the synthesis time of both compounds, from over 12 hours total dwell time to just two hours, while maintaining high purity of the target material. In the case of NCB, the product phase formed at a much lower temperature of 800 °C instead of 1100 °C than the seminal synthesis, resulting in a much more energy-efficient synthesis. It was found that, for the NADES synthesis method, it was necessary to ensure that the furnace was well-ventilated to obtain the ordered polymorph of NNB, though further annealing steps could also be utilised to convert the disordered polymorph into the ordered polymorph.

Additionally, SQUID magnetometry was performed on NCB for the first time, and it was found to exhibit very weak magnetism. It is possible that doping this material could yield some superconductive properties, which could be explored in future work. Finally, NNB was found to be antiferromagnetic with a Néel temperature of 11.9 K, which is consistent with previous work.

From direct comparison of the methods, it appears that for NCB the most consistent synthetic method is to use NADES. In contrast, for NNB, we found the most consistency with the aqueous method as it was more likely to form a product without stacking defects. In addition, the NADES method tended to form a porous product, whereas, for NNB, the aqueous synthesis yielded a more closed material.

With this work, we have enabled the facile synthesis of NCB, hopefully allowing its potential applications to be explored in the future. In addition, we anticipate that these synthesis techniques could also be applied to additional honeycomb layered metal oxides to NNB, enabling the rapid synthesis of the ordered polymorphs of this material in the future.

## Author contributions

S. R. H. initiated and supervised the project. E. J. L., J. P. performed the synthesis and structural characterisation experiments at Bristol. S. F. provided additional supervision and discussion. All authors contributed to the discussion of the results, analysis of the materials and to manuscript preparation.

## Conflicts of interest

There are no conflicts to declare.

## Supplementary Material
